# Innovative Techniques for Estimating Illegal Activities in a Human-Wildlife-Management Conflict

**DOI:** 10.1371/journal.pone.0053681

**Published:** 2013-01-16

**Authors:** Paul Cross, Freya A. V. St. John, Saira Khan, Andrea Petroczi

**Affiliations:** 1 School of the Environment Natural Resources and Geography, Bangor University, Bangor, United Kingdom; 2 Durrell Institute for Conservation and Ecology, University of Kent, Canterbury, Kent, United Kingdom; 3 Faculty of Science, Engineering and Computing, Kingston University, Kingston upon Thames, Surrey, United Kingdom; 4 Department of Psychology, The University of Sheffield, Sheffield, United Kingdom; University of Western Ontario, Canada

## Abstract

Effective management of biological resources is contingent upon stakeholder compliance with rules. With respect to disease management, partial compliance can undermine attempts to control diseases within human and wildlife populations. Estimating non-compliance is notoriously problematic as rule-breakers may be disinclined to admit to transgressions. However, reliable estimates of rule-breaking are critical to policy design. The European badger (*Meles meles*) is considered an important vector in the transmission and maintenance of bovine tuberculosis (bTB) in cattle herds. Land managers in high bTB prevalence areas of the UK can cull badgers under license. However, badgers are also known to be killed illegally. The extent of illegal badger killing is currently unknown. Herein we report on the application of three innovative techniques (Randomized Response Technique (RRT); projective questioning (PQ); brief implicit association test (BIAT)) for investigating illegal badger killing by livestock farmers across Wales. RRT estimated that 10.4% of farmers killed badgers in the 12 months preceding the study. Projective questioning responses and implicit associations relate to farmers' badger killing behavior reported via RRT. Studies evaluating the efficacy of mammal vector culling and vaccination programs should incorporate estimates of non-compliance. Mitigating the conflict concerning badgers as a vector of bTB requires cross-disciplinary scientific research, departure from deep-rooted positions, and the political will to implement evidence-based management.

## Introduction

The successful management of biological resources at local, national and international scales is contingent upon adherence to an effective system of rules that regulate the behaviours of stakeholders. Imperfect compliance can have detrimental impacts upon the environment as illustrated by illegal deforestation, pesticide use, and fishing [1], [2], [3], [4]. Illegal fish catches are known to be globally widespread holding profound consequences for the setting of appropriate quotas based on estimated maximum sustainable yield [5]. Difficulties associated with determining levels of non-compliance [6] can hold consequences for regulators, for example in the prevention of rabies through control of the movement of pets [7], [8]. There is limited evidence to suggest that policy initiatives incorporate sufficiently reliable non-compliance estimates when setting objectives [5]. This may be due in large part to the fact that estimating illegal activity directly, is inherently problematic as rule breakers are generally unwilling to reveal their activities due to concerns of retribution. Consequently, such data are highly prone to biases that can undermine the setting of appropriate management policy [9], [10].

The illegal killing of protected wildlife is a prominent example of a sensitive regulatory topic which is difficult to study directly; consequently reliable prevalence estimates are few. Globally, the expansion of human activities, combined with the restoration and legal protection of wildlife populations has led to increasing contact between humans and wildlife [11]. In many instances this has led to increased conflict including livestock depredation by carnivores [12], predation of game birds by raptors [13], and threats to human life [14]. Conflicts can create considerable controversy when legal issues are concerned and livelihoods are at risk; in such instances mitigation can be politically sensitive and political pressures may override scientific evidence [15]. This problem appears particularly acute with respect to free-ranging wildlife associated with disease transmission such as the European badger (*Meles meles*) [16], long associated with the transmission of bovine tuberculosis (bTB) to cattle [17], [18].

### Management of bovine tuberculosis

Management of the spread and transmission of the bacterium *Mycobacterium bovis*, the causative agent of bTB, has frequently focused on badger culling programmes [19], [20], [21]. The consequences of culling are epidemiologically complex, due in part to the social structure of badger populations and their territorial behaviour [22]. Controlling the disease in England over the last ten years has cost British tax payers £500 million [23].

The type of culling (proactive or reactive) has been shown to significantly influence the short-term success of disease control, the benefits of which do not necessarily translate into longer-term cost-effective disease control [24]. Proactive culling (widespread and repeated culling of badgers) in the Randomized Badger Culling Trial (RBCT) achieved moderate reductions in bTB infection of cattle within proactive cull areas [25], [26]. However, bTB incidence significantly increased in neighbouring areas (≤1.5 km outside proactive cull areas) [24]. This is consistent with findings from reactive cull areas (small-scale localised culling of badgers in response to specific bTB outbreaks) where the incidence of bTB increased [27]. The social disruption of badgers caused by localised culling (perturbation effect) has been shown in some studies to increase the home range area of badgers, increasing the opportunity for disease to spread [19]. The final report of the £48 million RBCT [27] acknowledged that non-compliance with trial operations, such as the illegal killing of badgers (especially in the survey only area of the RBCT), could have obscured study findings. The levels of illegal badger killing were not investigated by the RBCT; nor have levels been estimated by any other study and as such, remain unreported. However, the RBCT's authors considered the trial's statistical power sufficient to overcome any non-compliance encountered.

Knowledge of the existing prevalence of illegal badger killing may prove critical in understanding the effects of culling in controlling the spread of bTB. This study sets out to provide a robust estimate of illegal badger killing within the livestock farming community in Wales using a combination of innovative social science techniques which permit the indirect questioning of farmers.

### Estimating illegal behaviour

National laws prohibit the killing of badgers in the UK, except under license. Estimating the proportion of farmers illegally killing badgers using a conventional questionnaire approach is problematic as farmers may not respond honestly to explicitly incriminating questions. It is well understood that obtaining reliable estimates of illegal activities is inherently problematic [6], [28], as respondents may be unwilling to respond honestly to traceable questioning [5], [29]. Recent studies suggest that the validity of data on illegal behaviours is significantly improved using specialized methods such as the Randomized Response Technique (RRT) which provides respondents with high levels of privacy and anonymity, increasing the proportion of honest responses [30], [31]. In comparative studies, RRT has returned higher estimates of involvement in sensitive and illegal behaviours when compared to conventional anonymous surveys [29], [32].

Related studies suggest that respondents' characteristics, such as their attitudes, and estimates of their peers' behaviour (projective questioning), can indicate their own involvement in illicit behaviour [32], [33]. However, studies testing the effectiveness of such indicators by linking them to an actual measure of behaviour are scarce [34], [35].

Projective questioning (PQ) asks respondents about other peoples' behaviour instead of the respondents' own behaviour [36]. When the behaviour of interest is sensitive or illegal, evidence suggests that PQ estimates are biased by personal perceptions [36]. In some cases this ‘egocentric bias’ (coined the ‘False Consensus Effect’ [37]) occurs in respondents who endorse a socially questionable behaviour and can indicate the respondents' own involvement in illegal activity [37], [38], [39].

A further technique, the Brief Implicit Association Test (BIAT) [40] measures the relative strength of automatic associations between concepts by measuring the reaction time taken by respondents to categorise stimuli into pre-defined categories when they view them on a computer screen. The underlying notion of this task is that if a concept-attribute pair are highly associated (e.g. flowers+beautiful), participants will find this categorisation condition easier to identify and associate compared to an un-associated pair (e.g. flowers+ugly). The strength of association is evidenced by a speedier reaction time when these categories share the same response key on the computer keyboard, compared to different keys. If the two concepts which share the same response key are not associated with each other (e.g. flowers+ugly), reaction time is expected to be slower [40].

In this study RRT was used to estimate the proportion of farmers in Wales illegally killing badgers. Using a specialised form of logistic regression (van den Hout et al. 2007) the utility of PQ and BIAT as indirect indication of involvement in illicit behaviours are explored.

## Methods

### Ethics Statement

The study was approved by the College of Natural Science Ethics Committee at Bangor University, and conformed to the principles set out in the Declaration of Helsinki. Participants provided informed verbal consent, as approved by the ethics committee.

### Respondent sample

The survey (copy available from corresponding author) was undertaken between June and September 2011 at five major agricultural shows and 12 farmers' markets across Wales. A convenience sample of farmers (defined as any person farming livestock in Wales) self-completed one paper copy of the survey; only the BIAT section was administered via computer. Farmers encountered more than once were not re-surveyed. No personal identifying information was collected from farmers beyond gender, year of birth, county of residence and the first half of their postal code (e.g. LL57). Providing such anonymity facilitates respondent candour.

The survey did not require respondents to indicate whether they had culled badgers under license. This omission was based on prior knowledge that in 2010 the Welsh Assembly Government issued 12 licenses under section 10 of the Protection of Badgers Act 1992 for the removal or culling of badgers. No badgers were culled under these licenses in 2010, only removed. Consequently, it can be assumed that all reported badger killings in this study were illegal as decreed by the Protection of Badgers Act 1992.

### Randomized response technique

RRT introduces a randomizing device (such as dice) to the question-answer process increasing the level of protection perceived by respondents when asked to answer sensitive questions, the answers to which may be incriminating [31]. Depending upon the result of the randomizing device, respondents are instructed to either: answer a sensitive question truthfully (their answer can be ‘yes’ or ‘no’), or to answer ‘yes’, or ‘no’ irrespective of the truth as prescribed by instructions associated with the randomising device (Boruch 1971 in [31]). The RRT question contained in this survey followed such a ‘forced response’ design as applied by others [35],[41].

Respondents were required to roll two dice prior to answering the sensitive question ‘*In the last 12 months did you kill any badgers?*’. The sum total of the two dice determined whether respondents were required to answer the sensitive question honestly, or were ‘forced’ to answer ‘yes’ or ‘no’ irrespective of the truth. When the dice summed five through to ten, respondents were required to answer truthfully. When the sum of the dice was two, three, or four respondents were obliged to answer ‘yes’, and when the sum of the two dice equalled 11 or 12 respondents were obliged to answer ‘no’. Respondents rolled the dice in an opaque plastic beaker so that the dice score was visible only to them. Results of the dice roll were never revealed to researchers.

There are dual benefits of using two dice over only one. Firstly, efficiency is increased, as there is a 75% chance that the respondent will be required to answer the sensitive question honestly compared to a 66% chance with only one die [42]. The second is that the respondent, understandably, believes that he or she has a near 50% chance of rolling a forced (2, 3, 4, 11, 12) compared to an unforced score (5, 6, 7, 8, 9 and 10). The apparent near parity between scores for forced and honest responses provides the respondent with an augmented sense of protection in answering honestly. This perception is however incorrect as in reality respondents will roll one of the forced scores only 25% of the time. This increased response efficiency facilitates improved predictions of population level prevalence of a behaviour without the need to increase the sample size [30].

### Projective questioning and the false consensus effect

Projective questioning (PQ) builds on the assumption that people tend to know about the socially sensitive behaviours present or absent in their social group. However, estimates given on these behaviours tend to be part cognitive and part motivated egocentric perceptions, rather than objective accurate accounts [33], [37]. The term ‘False Consensus Effect’ was introduced to describe the phenomenon by which people project their own behaviours onto others, thus overestimating the prevalence of a given behaviour they are involved in or endorse [37]. Consequently, respondents' population-level estimates of other peoples' behaviour tend to be biased in accordance with their own behaviour [33]. For example, cigarette smokers estimate a higher proportion of smokers in the population compared to non-smokers [43]; students willing to make monetary and voluntary work contributions for environmental causes believed that a higher percentage of their classmates would also do so and vice versa [44]. To investigate the relationship between farmers' projective questioning estimates of badger killing and their own badger killing behaviour as reported via RRT, farmers were asked to state the proportion of farmers they believed to be killing badgers in response to the following question *‘Out of every 100 farmers, how many do you think have controlled badgers by killing in the last 12 months?’.* High estimates were expected to be related to admitting to killing badgers as determined by RRT estimates.

### Brief implicit association test (BIAT)

In this study, the BIAT [38] was used to examine if badgers (valence category) would be more strongly associated with a positive valence (nurture) or a negative valence (control). Lexical and pictorial stimuli of four categories were presented in each block but only two of the four categories were focal (i.e. associated with badgers and killing of badgers). The non-focal category comprised pictures of dogs. Participants were instructed to respond by pressing the ‘I’ key when they saw the focal categories, e.g. ‘badgers and control’ or ‘badgers and nurture’. When they saw anything else which fell outside of these categories, e.g. ‘dogs and nurture’ or ‘dogs and control’ they were asked to press the ‘E’ key.

The BIAT consisted of five blocks, one practice block and four test blocks with each combined-task presented twice. Each stimulus was presented at least once in the test blocks with some stimuli presented twice. Stimuli presented twice were randomly selected from the pool of all stimuli. In the practice block, only stimuli from the concept categories badgers and dogs were presented, each stimulus was presented twice with a few stimuli randomly selected to be presented three times. The order of the test blocks was counterbalanced between subjects, with half of the participants completing the BIAT in the following order: ‘badgers and nurture’, followed by ‘badgers and control’, ‘badgers and control’, ‘badgers and nurture’. The remaining half received the BIAT in the reverse order. The recommended procedure of repeating the BIAT so that each combined task is presented twice was followed to increase test reliability [38]. The BIAT measures the difference in reaction time between the two conditions (e.g. ‘badgers and control’ and ‘badgers and nurture’); the D-score obtained reveals the strength of association between the concepts and its interpretation is similar to Cohen's *d* [45]. The D-score can range between −2 and +2 revealing the strength of the association, where the closer the score is to −2 or +2 indicating a stronger automatic association. In this study, positive scores represent an automatic association between the categories ‘badgers’ and ‘nurture’ and negative scores represent an automatic association between ‘badgers’ and ‘control’. D-scores between 0 and −0.15 represent weak associations for ‘badgers and control’, −0.16 to −0.64 represent moderate associations, and −0.65 and below are considered strong associations [38], [40]. Low estimates were expected to relate to RRT estimates of admitting to killing badgers.

### Data analysis

The proportion of farmers killing badgers (RRT responses) was estimated using the model of Hox & Lensvelt-Mulders [46]:
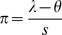
where *π* is the estimated proportion of the sample who have undertaken the behaviour, *λ* is the proportion of all responses in the sample that are ‘yes’, *θ* is the probability of the answer being a ‘forced yes’, and *s* is the probability of having to answer the sensitive question truthfully. Ninety-five per cent confidence intervals for RRT data were estimated from 10,000 bootstrap samples providing confidence intervals that incorporate both the uncertainty arising from the RRT and sample uncertainty. Significant differences between farm type (livestock kept), and the prevalence of badger killing was concluded when the bootstrapped 95% confidence intervals for the mean difference did not include zero.

The BIAT latency was measured in milliseconds (ms) and then transformed into D-scores. The D-scores were calculated using an optimised scoring algorithm [40] where trials with latencies (the elapsed time to response) above 10,000 ms were discarded and participants who had more than 10% of trials with latencies below 300 ms were removed. The number of errors were recorded and error trials were included using the built in penalty [40] where latencies were recorded until the correct response was provided.

Relationships between farmers' reported badger killing behaviour (RRT responses), their PQ estimates, and BIAT D-scores were investigated using generalized linear models (GLM) in R v. 2.15.0 [47]. The GLM used a customized link function incorporating the known probabilities of the forced RRT responses [35], [48]. To investigate the effectiveness of PQ estimates and BIAT D-scores at predicting badger killing behaviour GLMs incorporating either PQ estimates or BIAT D-scores were statistically compared (likelihood ratio test) to a null model. Finally, likelihood ratios were calculated from the fitted models.

## Results

A total of 428 farmers (2.87% of the total population of 14,917 cattle and sheep farmers in Wales) returned completed surveys, 150 of whom also completed the BIAT. The majority of farmers were male (77.8%, *n* = 333) and the mean age was 50 years (s.e. = 0.7, *n* = 425). Farmers stocked their farms with only sheep (40.9%, *n* = 175), only cattle (29.7%, *n* = 127), and cattle in combination with other livestock (26.6%, *n* = 114). The remaining 2.1% (*n* = 9) of farmers kept other types of livestock. The proportion of farmers reporting killing badgers in the twelve months prior to the study was 10.4% (95% CI: 5.1%, 15.7%). A higher, but not significant proportion of farmers stocking only cattle admitted to killing badgers (14.5%), compared to those stocking cattle and other livestock (12.8%; mean difference between cattle only and cattle and other livestock 1.6%), or sheep (6.7%; mean difference between cattle only and sheep only 7.9%).

Farmers' PQ estimates ranged from zero to 100% (mean = 10.3% std. dev. ±23.1, n = 428). The large variation and presence of PQ estimates up to 100% suggests that some farmers have FCE-biased views potentially indicating self-involvement. Results of the fitted GLM show that the likelihood of killing badgers was positively related to PQ estimates, indicating that as farmers' projective estimates increased so too did the likelihood of their admitting to killing badgers (via RRT) (Table 1). Compared to a null model, PQ estimates were a significant predictor of badger killing behaviour (likelihood ratio χ^2^ 12.9, p = 0.01, with df = 1). Odds ratios calculated from the fitted model indicate that farmers reporting PQ estimates of 100% (maximum value reported) were 3.17 times more likely to have reported (via RRT) killing badgers, compared with farmers reporting PQ estimates of zero (minimum value reported).

**Table 1:** Intercept and coefficient values of fitted generalized linear models incorporating either farmers PQ estimates or BIAT D-scores as predictors of badger killing behaviour as reported via RRT.

	Intercept	Coefficient	S. error	P value
Projective questioning	−2.41	0.02	0.007	<0.001
BIAT	−2.54	−0.96	1.43	0.51

The mean BIAT D-score (−0.38, std. dev. ±0.39, n = 150) indicates that farmers more readily associate badgers with killing (82.6%), rather than conserving. Twenty five percent (n = 38) of farmers strongly associated badgers with killing (BIAT D-score≤−0.65). The fitted GLM indicates that the likelihood of admitting to killing badgers was negatively related to BIAT scores showing that as farmers more strongly associated badgers with control, rather than nurture, they were more likely to have admitted (via RRT) to killing badgers (Table 1). Compared to a null model, BIAT D-scores were not a significant predictor of badger killing behaviour (likelihood ratio χ^2^ 0.8, p = 0.53, with df = 1). Odds ratios calculated from the fitted GLM indicate that farmers scoring the lowest D-score (−1.14) were 1.84 times more likely to have admitted (via RRT) to killing badgers compared to farmers scoring the highest D-score (0.70).

## Discussion

This study presents a baseline estimate of illegal badger killing at a national scale. The overall proportion of farmers admitting to killing badgers was 10.4%, with the highest proportion of illegal badger killing reported by farmers stocking only cattle (14.5%). RRT estimates represent a conservative estimate of badger killing across the study area and provide evidence that higher illegal killing rates could be expected on cattle-only farms. The finding that 6.7% of sheep-only farmers reported killing badgers is intriguing as there is no explicit reason for such behaviour. It may suggest a background level of badger killing for sport, or that farmers have a collective sense of responsibility to control badgers, particularly in regions where sheep and cattle farms share boundaries.

Due to the epidemiological complexities associated with the spread of bTB [19], it is beyond the scope of this study to suggest how the estimated prevalence of illegal badger killing (10.4%) would impact upon disease spread. However, our opinion is that a rate of 10% illegal killing would have a non-negligible impact. Beyond epidemiological complexities, understanding the potential impacts upon disease spread is further complicated by the likelihood that illegal killing may vary in intensity, over time [49], and between regions and livestock systems. Further, such illegal activity may also vary between areas of high and low bTB prevalence. Future studies should attempt to fully evaluate the significance of illegal killing as a driver of disease spread, particularly when investigating the relative advantages of different vaccination and culling regimes, such as those proposed for Wales and England in the coming years.

There is considerable evidence that RRT provides more accurate estimates of sensitive behaviours compared to conventional survey methods [28], [31], [50]. However, this comes at a cost. RRT requires larger samples compared to conventional techniques in order to obtain estimates with acceptable levels of error [51]. Larger sample sizes require a contingent increase in research costs. However, we suggest that increased costs are compensated for by the corresponding increase in data validity [30].

By using a customized link function [35], [48] the logistic regression model was adapted in order to investigate the relationship of indirect measures of behaviour (PQ estimates and BIAT D-scores) with our ‘best-measure’ of farmers' involvement in illegal badger killing captured by RRT. In our first fitted model PQ estimates were positively related to RRT response; as farmers' estimates of their peers' badger killing behaviour increased, as too did the probability that they themselves admitted to killing badgers. This finding supports the existence of the false consensus effect [37], [52] and the suggestion made by others [34], [35] that asking respondents about their peers' behaviour, may be a useful way of identifying groups of people involved in socially undesirable behaviours.

The second fitted model explored the relationship between farmers' badger killing behaviour (as reported via RRT) and their implicit attitudes towards badgers (BIAT D-scores). BIAT D-scores were negatively related to farmers' RRT responses, indicating that as farmers' propensity to associate badgers with ‘control’ increased, so too did the probability that they had admitted to killing badgers. However, the calculated odds ratios suggest that projective questioning is more useful than BIAT at distinguishing between farmers who are more or less likely to have killed badgers.

Implicit associations create a propensity for the behaviour in question, but its effect on behaviour-implementation is moderated by other individual and situational factors. For instance, research over the past two decades has demonstrated that feelings and motivations driven by our consciousness constitute but a minor segment of our inner thoughts. Processes outside conscious awareness or control exert significant influence on perception, judgement, and consequently actions [53]. Studies have linked implicit associations to future behaviours ranging from job-related decisions [54], consumer choice decisions [55], [56] and suicide attempts [57]. A meta-analysis of 122 independent reports [58] found evidence for moderate but statistically significant relationships between implicit associations and behaviours, where implicit associations explained a proportion of variance in behaviour over and above self-report measures. Explanatory variables included differences between individuals [59], motivation and opportunity [56], as well as self-control [60], suggesting that implicit associations can help shape behaviours.

Convenience sampling was used to recruit farmers to the study. Given that convenience sampling is non-random it has the potential to introduce bias to surveys [61]. However, as every possible farmer encountered at study sites was approached in the time available we consider this potential source of bias to be negligible. Farmers were not asked if they had suffered a recent bTB breakdown in their herd. This represents a potentially missed opportunity as such information could have been used to explore experiential drivers of illegal badger killing behaviour.

Findings from the RBCT appear to suggest a critical culling-intensity of between 50% and 100% of badgers in an area, where if too few badgers are culled then the risk of increased disease spread appears possible (based upon the reported findings of the RBCT reactive culling data) [26], [62]. Incorporating estimates of illegal killing of badgers, as reported in this study, would allow the parameters for critical culling intensity to be refined. In turn, this would provide more accurate data to inform subsequent policy decisions aimed at reducing the prevalence of bTB in cattle. Research designed to evaluate the efficacy of mammal vector culling and vaccination programs should incorporate estimates of non-compliance with rules that may impact upon study findings.

This study provides further evidence of the utility of RRT as a method for investigating sensitive topics, and projective questioning as an indicator of people's involvement in illicit acts. Mitigation of the conflict concerning badgers as a vector of bTB, farmers, and those who represent them, requires evidence from cross-disciplinary research. To this end much has been achieved. However, scientific evidence is insufficient where political will to implement evidence-based management is lacking and the entrenched position of stakeholders presents a barrier to effective conflict mitigation [15].
